# Insights into Metal Oxide and Zero-Valent Metal Nanocrystal Formation on Multiwalled Carbon Nanotube Surfaces during Sol-Gel Process

**DOI:** 10.3390/nano8060403

**Published:** 2018-06-05

**Authors:** Dipesh Das, Indu V. Sabaraya, Tara Sabo-Attwood, Navid B. Saleh

**Affiliations:** 1 Department of Civil, Architectural and Environmental Engineering, The University of Texas at Austin, Austin, TX 78712, USA; dipesh.das@utexas.edu (D.D.); indu.venu@utexas.edu (I.V.S.); 2 Department of Environment and Global Health, University of Florida, Gainesville, FL 32610, USA; sabo@phhp.ufl.edu

**Keywords:** nanohybrid, hybridization, standard electron potential, crystal phase, reducing agents

## Abstract

Carbon nanotubes are hybridized with metal crystals to impart multifunctionality into the nanohybrids (NHs). Simple but effective synthesis techniques are desired to form both zero-valent and oxides of different metal species on carbon nanotube surfaces. Sol-gel technique brings in significant advantages and is a viable technique for such synthesis. This study probes the efficacy of sol-gel process and aims to identify underlying mechanisms of crystal formation. Standard electron potential (SEP) is used as a guiding parameter to choose the metal species; i.e., highly negative SEP (e.g., Zn) with oxide crystal tendency, highly positive SEP (e.g., Ag) with zero-valent crystal-tendency, and intermediate range SEP (e.g., Cu) to probe the oxidation tendency in crystal formation are chosen. Transmission electron microscopy and X-ray diffraction are used to evaluate the synthesized NHs. Results indicate that SEP can be a reliable guide for the resulting crystalline phase of a certain metal species, particularly when the magnitude of this parameter is relatively high. However, for intermediate range SEP-metals, mix phase crystals can be expected. For example, Cu will form Cu_2_O and zero-valent Cu crystals, unless the synthesis is performed in a reducing environment.

## 1. Introduction

Carbon nanotube–metal nanohybrids (NHs) are being considered for large scale use as electro- and photo-catalysts [1] and are studied for electronics [2], gas sensing [3], biosensing [4], and laser [5] applications. With the increased commercial value, the bulk synthesis of these NHs is attracting interest. A simple sol-gel technique can be a viable process that can produce 100s of mg of multiwalled carbon nanotube (MWNT)–metal NHs [6]. Both zero-valent and oxides of metals can be formed on MWNT surfaces. However, the choice of the metal and its inherent electronic properties will dictate the resulting crystalline phases. Since preserving the oxidation state of the metal crystals is crucial to render their reactive properties [7,8], understanding the mechanism of nanocrystal formation with a particular crystal phase is thus necessitated.

When preparing metallic nanomaterials, achieving a high degree of crystallinity (of the synthesized materials) is essential to extract the desired optical, electronic, and chemical properties [9]. The rate of nucleation during crystal formation on a surface is a strong function of the surface energy as well as the thermodynamic driving force. The former (i.e., the surface energy) is influenced by the surface moieties (e.g., carboxyl groups on crystalline or polymeric [10,11,12] materials), and the latter (i.e., the thermodynamic driving force) [13], which is described as the difference in Gibbs free energy between the crystal phase and the surrounding liquid, is a function of the precursor amount present during synthesis. Thus, the synthesis methods and operating conditions (e.g., temperature [14], reducing agent [15], and precursor amount [13]) are critical elements that need to be adjusted appropriately for the preparation of metal nanocrystals with a high degree of crystallinity [14] and a desired redox state in the metal species [16]. Calcination can facilitate the preparation of ordered structures, but the feasibility of applying such a high temperature of 500 °C or higher can be limited when carbon nanotubes are involved in the mix [6,17]. The chemical attachment of metallic nanocrystals can facilitate MWNT oxidation and lower the MWNT oxidation temperature via the chemical modification of the MWNT surface. [6] However, such processes are conducive to oxide formation; hence, synthesizing zero-valent nanocrystals can be challenging.

In sol-gel synthesis, strong reducing agents (e.g., borohydride salts) are typically added to form zero-valent nanocrystals [15,18]. However, the addition of reducing agents drives the reaction toward zero-valent metal formation (rather than chemical attachment), which leads to isolated and unassociated (from MWNTs) nanocrystal formation. The excess unassociated metal particles then require rigorous post-treatment of the materials to separate the NHs from the unattached nanocrystals. Furthermore, some of the metals, because of the elemental electron properties, present further challenges in zero-valent metal crystal formation.

The standard electron potential (SEP) of a metal species can dictate the reaction pathway, and hence can control the oxidation state (i.e., metal vs. metal oxide) of the crystal grown on MWNT surfaces. SEP values represent electron transfer capabilities between the oxidized and the zero-valent metal forms (i.e., M*^n^*^+^ + Ne^−^ ↔ M, where M is the metal species and *n* is the number of electrons involved in the exchange). Literature evidence suggests that metals with negative SEP preferentially form oxides while those with positive values tend to form zero-valent forms of the same. Metal species that are commonly reported to form oxides on carbon nanotube surfaces possess strongly negative SEP values (Table S1). The following oxides are reported to have formed with metals: Al_2_O_3_ [19,20,21], CeO_2_ [22,23], CoO_3_ [24,25], Eu_2_O_3_ [26,27], Fe_x_O_y_ [28,29,30,31], HfO_2_ [32,33], MgO [34], MoO_2_ [35], NiO [36], SiO_2_ [37,38,39], SnO_2_ [40], TiO_2_ [41,42], V_x_O_y_ [43], ZnO [44], and ZrO_2_ [45]. On the other hand, Ag [46], Au [47], Pt [48], and Pd [49] with a positive SEP are reported to form zero-valent metals on Carbon Nanotube (CNT) surfaces. Cu and W (with positive SEP) and Fe (with negative SEP) are exceptions to this trend; i.e., despite their positive SEP values, Cu [50] and W [51] are shown to form oxides, whereas Fe with a negative SEP can form zero-valent metal nanocrystals [52]. The challenge, however, is to comment on the role of SEP on forming zero-valent vs. oxide crystals when the sol-gel method is employed to synthesize MWNT-based NHs.

This article aims to evaluate the efficacy of sol-gel process for in situ formation of metal vs. metals oxides onto MWNT surfaces with no extra addition of reducing or oxidizing agents. The study judiciously choses three metal species, namely Zn, Ag, and Cu; Zn and Ag has strong negative and positive SEP values (Zn with -0.763 V and Ag with +0.799 V SEP values), respectively, while Cu lies in the positive range, but with a much lower magnitude (SEP of +0.345 V) compared to Ag. Transmission electron microscopy is used to evaluate the NH morphology, while X-ray diffraction (XRD) is utilized to characterize the materials before and after calcination. The design of the study is carefully carried out (e.g., synthesizing and characterizing in absence of air to avoid oxidation) and tests the efficacy of sol-gel method to form nanocrystals with both types of crystal phases. 

## 2. Materials and Methods

### 2.1. Chemicals and Reagents

Pristine MWNTs (O.D. 8–15 nm) were procured from Cheap Tubes Inc. (Brattleboro, VT, USA). Concentrated nitric acid, sulfuric acid, and copper (II) nitrate monohydrate were purchased from Sigma Aldrich (St. Louis, MO, USA). Trace metal grade silver nitrate was purchased from Alfa Aesar (Haverhill, MA, USA). Isopropanol and dimethylformamide (DMF) were obtained from Fisher Scientific (Pittsburgh, PA, USA) while zinc (II) nitrate hexahydrate was purchased from J.T Baker (Center Valley, PA, USA). For preparing all aqueous suspensions and solutions, 18.2 mΩ (Milli-Q) water was used unless otherwise stated.

### 2.2. Nanohybrid Synthesis

All materials were synthesized using a modified sol-gel method [6]. In brief, MWNTs (1 g) were acid-etched by ultrasonication (Qsonica LLC, Newtown, CT, USA) in 300 mL of concentrated nitric and sulfuric acid mixture (1:1 volume basis). Upon sonication, the mixture was refluxed at 100 °C for 3 h under continuous stirring. The oxidized MWNTs were subsequently filtered until the pH of the filtrate reached >5.5 and then were dried for 48 h in a desiccator. After drying, the oxidized MWNTs were re-suspended in isopropanol with an ultrasonic dismembrator (Qsonica, Newtown, CT, USA) and transferred into a round bottom flask. Appropriate amounts, i.e., 123 mg of Zn, 85 mg of Cu and 71 mg of Ag precursors, were added to 10 mL of isopropanol and introduced drop wise to the MWNT-isopropanol suspension at 0.301 mL/min with a peristaltic pump (Ismatec, Wertheim, Germany). The slow rate of precursor addition was maintained to provide sufficient mixing time. The entire suspension was refluxed at 80 °C for 3 h in a nitrogen environment. Water was added drop wise into the reaction vessel to promote hydrolysis, where necessary. Afterwards, the refluxed mixture was washed 4 times with isopropanol (as a purification step), which removed any unreacted reagent. Finally, isopropanol was evaporated, the dry materials were powdered using a mortar and pestle, and the resultant materials were calcined at 400 °C for 3 h under nitrogen to facilitate crystal formation. 

### 2.3. Physical Morphology and Elemental Composition

The physical morphology of the NMs was determined using a JEOL 2010F high resolution transmission electron microscopy (HRTEM, JEOL, Tokyo, Japan) equipped with energy dispersive spectroscopy (EDS). Electron micrographs were obtained at an acceleration voltage of 200 kV. The details of the HRTEM methodology are described elsewhere [6,53,54,55,56,57,58,59]. In brief, drops of aqueous dispersions of NHs were placed on lacey carbon coated copper TEM grids (SPI Supplies, West Chester, PA, USA) and air-dried over a few minutes. Several micrographs were taken to obtain representative images. 

The elemental composition of the dry MWNT and NH samples was evaluated with a Kratos X-ray Photoelectron Spectrometer-Axis Ultra DLD, equipped with a monochromated Al K_α_ X-ray source (1.486 KeV) and a concentric hemispherical analyzer [6]. A thin layer of powdered sample was placed on a double-sided copper taped stainless steel bar. The bar was then placed in the analysis chamber and degassed for at least 3 h. The X-ray photoelectron spectroscopy (XPS) analysis was then performed to obtain the survey spectra as well as the spatial high-resolution spectra and the data was analyzed by fitting the high-resolution element specific peaks with CasaXPS software (Version 2.3.19). To ensure reproducibility and overall homogeneity, a total of 9 samples for each material (MWNT and three NHs) were analyzed (3 samples each in triplicate batches for all NHs).

### 2.4. Analysis of Crystallinity

The crystallinity of the metal oxide (MO) on the NH surfaces was evaluated with an XRD. A 600 W Rigaku MiniFlex 600 (Rigaku, Tokyo, Japan) with a Cu–Kα irradiator (0.154 nm wavelength) and a graphite monochromator was used at a step width of 0.02° (between 2θ values of 20° to 60°) and a scanning rate of 2°/min. For MWNT–Cu/Cu_2_O samples, the samples were inserted into an airtight XRD sample holder under vacuum before their measurement. This method for the XRD of MWNT–Cu/Cu_2_O samples was carried out in order to eliminate air exposure of the materials while performing XRD on them. The scattering was detected using a scintillation counter.

### 2.5. Measuring Oxidation-Reduction Potentials (ORPs)

ORPs were measured with a portable ultrameter (Myron L Company, Carlsbad, CA). Two reaction mixtures, i.e., MWNT + isopropanol + Cu (NO_3_)_2_·H_2_O and MWNT + DMF + Cu (NO_3_)_2_·H_2_O were heated to 70 °C for 1 h. After calibrating the ultrameter, 1 mL of the samples was placed in the ORP measurement chamber separately and the ORP was recorded.

## 3. Results and Discussion

### 3.1. Physical Morphology and Composition

**Figure 1:**
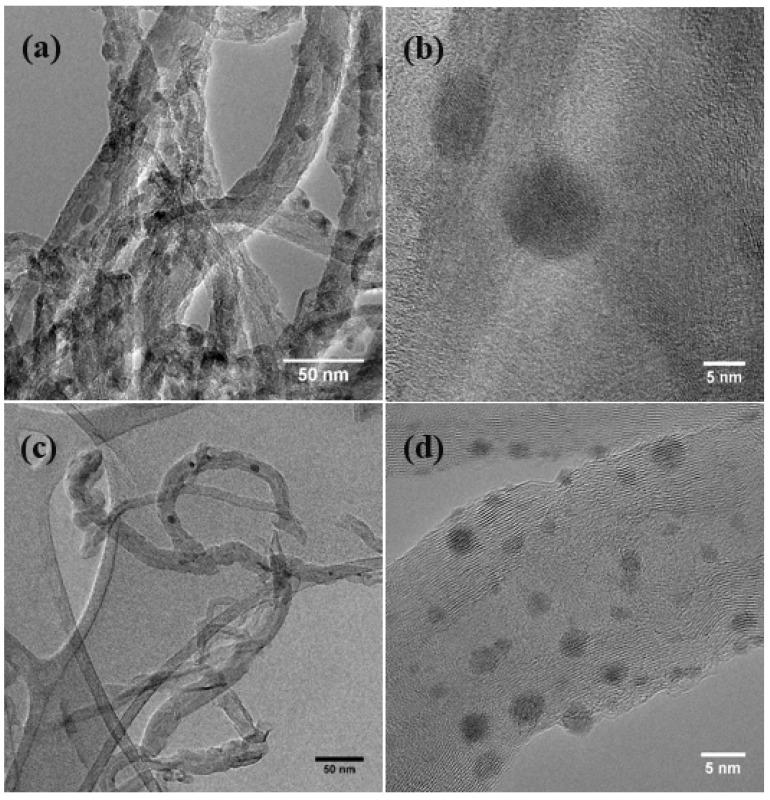

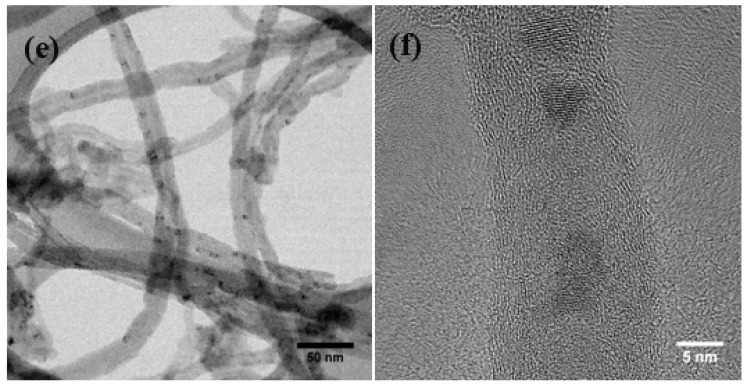
Representative TEM micrographs of (**a**,**b**) multiwalled carbon nanotube (MWNT)–ZnO, (**c**,**d**) MWNT–Ag, and (**e**,**f**) MWNT–Cu/Cu_2_O nanohybrids (NHs). High-resolution images are shown in (**b**,**d**,**f**).

Representative TEM micrographs of the NHs show tubular structures with spherical features (darker contrast) on the tubes (Figure 1). The higher magnification images (i.e., Figure 1b,d,f) show lattice fringes on the sphere-like features, indicating crystalline structures, while the exterior walls of the MWNTs are also observed in these images. The size of the nanocrystals is found to be larger for both the oxides (i.e., 8–10 nm for ZnO and 5–8 nm for Cu_2_O); the zero-valent crystals are smaller (i.e., 2–4 nm) and also are higher in density on the MWNT–Ag NH surfaces. These features are found to be distributed along the tubes. The composition of the samples is quantified with XPS, which indicates a stronger presence of the zero-valent metal compared to the oxides (Table S2). 

### 3.2. Chemical Attachment of Zn onto MWNTs: Hydroxide to Oxide Formation Pathway

**Figure 2:**
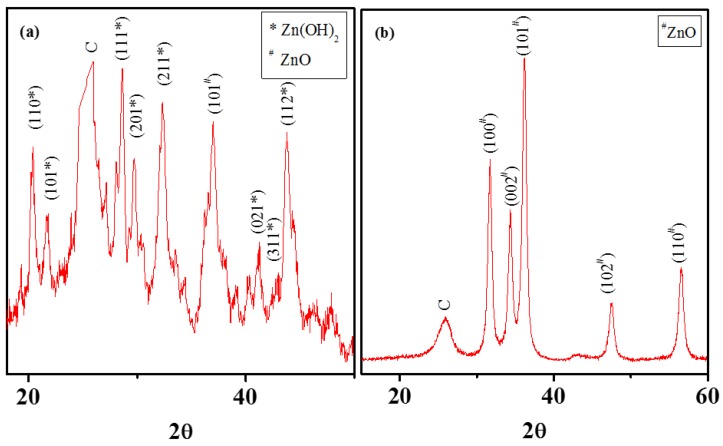
Representative XRD spectra of MWNT–ZnO NH (**a**) before and (**b**) after calcination at 400 °C for 3 h. The peak positions are labeled to indicate the respective crystal planes. The XRD spectra were collected at a scanning rate of 2°/min.

Nanocrystals growth on the MWNT surfaces was promoted by the negatively charged oxygen moieties on MWNT surfaces. Electrostatic attraction between metal cations and anionic surface moieties on MWNTs associate the Zn^2+^ with the MWNT surfaces. These ions then react with water molecules (generated from the hydrated zinc nitrate salt) to form Zn(OH)_2_ on the MWNT surfaces, which serve as nucleation sites for further growth of amorphous and mixed-phased Zn(OH)_2_ and ZnO. Nanocrystal formation pathway for MWNT-ZnO is evaluated in this study with XRD characterization on the materials, before and after calcination (Figure 2). XRD spectra before calcination shows evidence of both the crystal phases (Figure 2a). During calcination at elevated temeperature (at 400 °C in this case), the Zn(OH)_2_ likely loses the excess water and gets converted to ZnO crystal phases. XRD spectrum on the NH after calcinaton shows no evidence of Zn(OH)_2_ phase (Figure 1b) and confirms this likely crystal formation pathway. Literature reports on XRD patterns for amorphous Zn(OH)_2_ and ZnO are used to relate peak positions with specific crystalline planes [60]. The likely reaction pathway for MWNT-ZnO NH formation is shown below, which is similar to crystal formation pathway described for TiO_2_ growth on MWNTs [41].

$$O-{\mathrm{MWNT}}^{-}\overset{\mathrm{Zn}({\mathrm{NO}}_{3}){}_{2}}{\to }O-{\mathrm{MWNT}}^{-}{\mathrm{Zn}}^{2+}\overset{H_{2}O}{\to }\mathrm{MWNT}-\mathrm{ZnO}/\mathrm{Zn}{\left(\mathrm{OH}\right)}_{2}\left(\mathrm{amorphous}\right)\;\overset{\mathrm{Heat}}{\to }\mathrm{MWNT}-\mathrm{ZnO}\left(\mathrm{crystalline}\right)$$

### 3.3. Zero-Valent Metal Formation on MWNTs with no Reducing Agent

**Figure 3:**
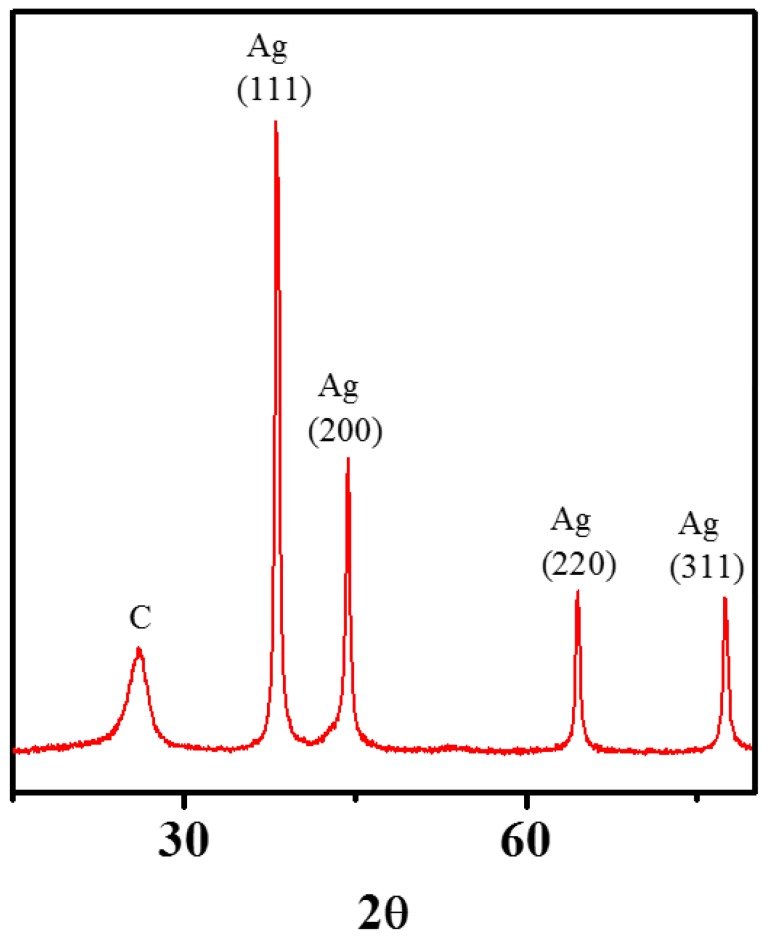
Representative XRD spectra of CNT–Ag NHs. The peak positions are labeled to indicate the respective crystal planes. The spectrum was collected at a scanning rate of 2°/min.

MWNT surfaces have successfully been enhanced with zero-valent Ag (with SEP of +0.799) crystals, employing the modified sol-gel method. It is noteworthy that no additional reducing agent was required for this synthesis. The XRD spectrum of the MWNT-Ag NH (Figure 3) shows defined peaks at (111), (200), (220), and (311) crystal planes, which correspond to zero-valent Ag [61]. Earlier studies on large-scale MWNT-Ag synthesis though report high quantity of Ag-attachment to MWNTs, the XRD spectrum show less-defined peaks, compared to the results presented in this study [46]. Though this study formed Ag-crystals on poly(acrylic acid)-modified MWNT surfaces, thus the underlying mechanism of these nanocrystal growth is likely quite different compared to those grown on oxidized carbon surfaces. 

### 3.4. Intermediate SEP-Metal Cu: The Anomaly That Forces Oxide Formation

**Figure 4:**
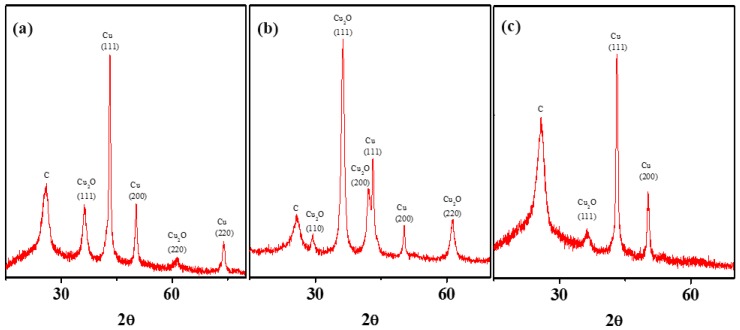
XRD spectrum of MWNT–Cu/Cu_2_O NH synthesized using the sol-gel process (**a**) in isopropanol, (**b**) in oxygen-free conditions with isopropanol, and (**c**) in dimethylformamide (DMF). An airtight XRD sample holder was used for XRD analysis for all the three materials. The peak positions are labeled to indicate the respective crystal planes. The XRD spectra were collected at a scanning rate of 2°/min.

With a positive SEP value, much like Ag, Cu should form zero-valent metals. However, Cu exhibits anomalous character and produces oxides during chemical attachment with MWNTs. This section attempts to overcome such oxide-forming propensity by using anoxic synthesis and characterization conditions, and results continue to be surprising. Following similar synthesis conditions (when compared to Zn and Ag), the Cu attachment resulted in a mixed Cu and Cu_2_O phases as shown in the XRD spectrum (Figure 4 a). Defined peaks at (111) and (200) planes (representing zero-valent Cu) and and at (220) and (111) (representing Cu_2_O phase) are consistent with the reported literature [62]. Literature reports on nano-scale zero-valent copper suggest that such behavior can stem from unavoidable oxidation during XRD characterization [50,51]. Some literature evidences also suggest that such XRD patterns are typical for Cu/Cu_2_O core/shell nanocrystals [62].

To facilitate zero-valent Cu formation on MWNTs, synthesis conditions were modified to avoid presence of ambient oxygen during the reaction (where, MWNT-isopropanol suspension was purged with nitrogen for 1 h and sampling handling was done in a glove box) and calcination processes. The synthesized NHs were also transferred into an airtight XRD sample holder to continue to avoid exposure to oxygen to the synthesized NHs. It is interesting to note that the nanocrystals formed in such anoxic reaction environment, continue to display Cu_2_O crystal planes, and with some additional Cu_2_O planes in higher intensity (Figure 4b). The results indicate that the likely oxidation of Cu has taken place, not during the XRD characterization, but likely during the synthesis process. The source of oxygen is likely H_2_O or NO_3_^-^, which could not be removed after the completion of the reaction process. These results indicate that the use of a reducing agent may be unavoidable for the lower magnitude SEP-metal Cu. 

A solvent with a relatively higher reduction potential (compared to isopropanol), e.g., dimethyl formamide (DMF), can potentially facilitate formation of zero-valent Cu in a sol-gel synthesis; earlier studies have employed DMF for synthesizing Ag nanoparticles [63]. Following similar protocol as noted earlier (in typical oxic environment), the nanocrystals formed with the aid of DMF exhibit a lowering of the (111) Cu_2_O peak, while a complete elimination of the (220) peak observed earlier (Figure 4c). To assess the reducing potency of the solvents ORP can be measured. The isopropanol system has an ORP value of +597 mV compared to DMF’s +504 mV; which indicate a more conducive reducing environment when DMF is used [64]. These findings strongly suggest that formation of zero-valent crystals with a sol gel method may be challenging for metals with low magnitude SEP, and may necessitate stronger reducing environment to facilitate this process.

## 4. Conclusions

Sol-gel synthesis can be utilized to form both zero-valent and oxides of metals on MWNT surfaces. The resulting crystal phase is strongly dependent on the electronic properties of the metal species. The SEP, which is a measure of energy required per unit charge to drive a redox reaction, can be used as a guideline for the choice of metal to obtain nanocrystals with the desired crystalline phase. Results suggest that metals with higher SEP values form either zero-valent or oxide phases, while those with lower magnitude SEPs facilitate mixed-phase crystals. The sol-gel technique can be useful to form zero-valent crystals without any reducing agent; however, such a reducing environment may become necessary for oxidation-prone metals such as Cu.

## Supplementary Materials

The following are available online at http://www.mdpi.com/2079-4991/8/6/403/s1.
