# Supplementary material for: Insights into Metal Oxide and Zero-Valent Metal Nanocrystal Formation on Multiwalled Carbon Nanotube Surfaces during Sol-Gel Process
Source: Nanomaterials (Basel). 2018 Jun 5;8(6):403. doi: 10.3390/nano8060403 (PMC6026900; doi:10.3390/nano8060403)
Supplement: Supplementary file 1 [file nanomaterials-08-00403-s001.pdf]

nanomaterials

Supplementary Information

Insights into Metal Oxide and Zero-Valent Metal Nanocrystal Formation on Multiwalled Carbon Nanotube Surfaces during Sol-gel Process

Dipesh Das [1], Indu V. Sabaraya [1], Tara Sabo-Attwood [2] and Navid B. Saleh [1,*]

[1] Department of Civil, Architectural and Environmental Engineering, The University of Texas at Austin, Austin, TX 78712, USA; dipesh.das@utexas.edu (D.D.); indu.venu@utexas.edu (I.V.S.)
[2] Department of Environment and Global Health, University of Florida, Gainesville, FL 32610, USA; sabo@phhp.ufl.edu
* Correspondence: navid.saleh@utexas.edu; Tel.: +1 (512)-471-9175

Table S1. Standard Electrode potential of different metal species: Reactions and values[1]

[1]Vanysek, P., Electrochemical series. *CRC handbook of chemistry and physics* **1998,** *87*

NH	Reaction	Standard Electrode Potential (V)
MWNT-Al_2O_3	Al^{3+} +3e$^-$ ↔ Al (s)	-1.662
MWNT-CeO_2	Ce^{3+} +3e$^-$ ↔ Ce (s)	-2.336
MWNT-CoO_3	Co^{2+} +2e$^-$ ↔ Co (s)	-0.28
MWNT-Cu_2O	Cu^{2+} +2e$^-$ ↔ Cu (s)	+0.345
MWNT-Er_2O_3	Er^{3+} +3e$^-$ ↔ Er (s)	-2.331
MWNT-Eu_2O_3	Eu^{3+} +3e$^-$ ↔ Eu (s)	-1.991
MWNT-Fe_xO_y	Fe^{2+} +2e$^-$ ↔ Fe (s)	-0.44
MWNT-HfO_2	Hf^{4+} +4e$^-$ ↔ Hf (s)	-1.55
MWNT-MgO	Mg^{2+} +2e$^-$ ↔ Mg (s)	-2.372
MWNT-MnO	Mn^{2+} +2e$^-$ ↔ Mn (s)	-1.185
MWNT-MoO_2	Mo^{3+} +3e$^-$ ↔ Mo (s)	-0.200
MWNT-NiO	Ni^{2+} +2e$^-$ ↔ Ni (s)	-0.25
MWNT-Pr_6O_{11}	Pr^{3+} +3e$^-$ ↔ Pr (s)	-2.353
MWNT-SiO_2	--	
MWNT-SnO_2	Sn^{2+} +2e$^-$ ↔ Sn (s)	-0.1375
MWNT-TiO_2	Ti^{2+} +2e$^-$ ↔ Ti (s)	-1.63
MWNT-V_xO_y	V^{2+} +2e$^-$ ↔ Pr (s)	-1.13
MWNT-WO_3	W^{3+} +3e$^-$ ↔ W (s)	+0.1
MWNT-ZnO	Zn^{2+} +2e$^-$ ↔ Zn (s)	-0.7628
MWNT-ZrO_2	Zr^{4+} +4e$^-$ ↔ Zr (s)	-1.45
MWNT-Ag	Ag^{+} +e$^-$ ↔ Ag (s)	+0.7996
MWNT-Au	Au^{3+} +3e$^-$ ↔ Au (s)	+1.498
MWNT-Pd	Pd^{2+} +2e$^-$ ↔ Pd (s)	+0.951
MWNT-Pt	Pt^{2+} +3e$^-$ ↔ Pt (s)	+1.18

Table S2 Atomic percentages and molar ratio of carbon: metal estimated from the XPS spectra using CasaXPS software.

Sample	%C	%Metal
MWNT-ZnO	78.5±1.0	6.2±0.5
MWNT-Ag	89.4±0.9	8.3±0.5
MWNT-Cu/Cu2O	79.2±1.2	5.2±0.4
